# Enzymatically amplified linear dbDNA^TM^ as a rapid and scalable solution to industrial lentiviral vector manufacturing

**DOI:** 10.1038/s41434-022-00343-4

**Published:** 2022-05-24

**Authors:** Maria Barreira, Claire Kerridge, Sara Jorda, Didrik Olofsson, Alexander Neumann, Helen Horton, Sarah Smith-Moore

**Affiliations:** 1 Touchlight Genetics Ltd, Hampton, TW12 2ER United Kingdom; 2 Omiqa Bioinformatics GmbH, Altensteinstraße 40, 14195 Berlin, Germany; 3 Present Address: Cell and Gene Therapy Catapult, Guy’s Hospital, London, SE1 9RT United Kingdom; 4 Present Address: Medical Research Institute La Fe, 46026 Valencia, Spain

**Keywords:** Gene therapy, Nucleic-acid therapeutics

## Abstract

Traditional bacterial fermentation techniques used to manufacture plasmid are time-consuming, expensive, and inherently unstable. The production of sufficient GMP grade material thus imposes a major bottleneck on industrial-scale manufacturing of lentiviral vectors (LVV). Touchlight’s linear doggybone DNA (dbDNA^TM^) is an enzymatically amplified DNA vector produced with exceptional speed through an in vitro dual enzyme process, enabling industrial-scale manufacturing of GMP material in a fraction of the time required for plasmid. We have previously shown that dbDNA^TM^ can be used to produce functional LVV; however, obtaining high LVV titres remained a challenge. Here, we aimed to demonstrate that dbDNA^TM^ could be optimised for the manufacture of high titre LVV. We found that dbDNA^TM^ displayed a unique transfection and expression profile in the context of LVV production, which necessitated the optimisation of DNA input and construct ratios. Furthermore, we demonstrate that efficient 3’ end processing of viral genomic RNA (vgRNA) derived from linear dbDNA^TM^ transfer vectors required the addition of a strong 3’ termination signal and downstream spacer sequence to enable efficient vgRNA packaging. Using these improved vector architectures along with optimised transfection conditions, we were able to produce a CAR19h28z LVV with equivalent infectious titres as achieved using plasmid, demonstrating that dbDNA^TM^ technology can provide a highly effective solution to the plasmid bottleneck.

## Introduction

In recent years, lentiviral vectors (LVV) have demonstrated their enormous potential in the treatment of genetic diseases such as ß-thalassaemia [1, 2], sickle cell disease [3], adenosine deaminase severe combined immunodeficiency [4], and Wiskott–Aldrich syndrome [5]. Their widespread use in the manufacturing of chimeric antigen T cell (CAR-T) therapies, which have demonstrated striking clinical success in patients with B-cell malignancies [6–8], further underlines the importance of developing cost-effective and scalable platforms for LVV manufacturing. Considerable efforts have been made to engineer stable producer cell lines; however, these methods can be time-consuming, costly, and challenging due to the toxicity of certain lentiviral proteins and the difficulty of maintaining the expression of all necessary plasmids through expansion [9]. Manufacturing of LVV is therefore still largely dependent on transient plasmid transfection. Traditional bacterial fermentation techniques used to produce plasmid DNA are slow, expensive, and limited by a lack of manufacturing capacity. The production of sufficient quantities of GMP plasmid to support industrial manufacturing of LVV therefore imposes a bottleneck in the development of these therapies and precludes their application to diseases with large patient populations [10].

As an alternative to plasmid, Touchlight’s doggybone DNA (dbDNA^TM^) is an enzymatically amplified DNA vector produced with exceptional speed and a small footprint, enabling industrial-scale manufacturing in a fraction of the time required for plasmid. dbDNA^TM^ are minimal, double stranded, and covalently closed DNA vectors amplified through an in vitro dual enzyme process, with demonstrated utility in the production of viral vectors [11, 12], cell therapies [13, 14], and DNA vaccines [15, 16]. The enzymatic amplification process means that dbDNA^TM^ is completely free from any bacterial propagation elements and antibiotics. Furthermore, the use of high fidelity and highly processive phi29 polymerase for rolling circle amplification has the added benefit of enabling the amplification of long and complex DNA sequences that have proven difficult to amplify via bacterial fermentation. These factors combined make dbDNA^TM^ an ideal vector for use in gene therapy applications and a highly effective solution to the plasmid bottleneck.

We have previously demonstrated that dbDNA^TM^ can be used to produce functional LVV in a triple transfection production system [12, 17]. LVV derived from dbDNA^TM^ produced comparable transgene expression to plasmid-derived LVV in vitro, and neonatal mice that received dose-matched intracerebroventricular injections of LVV produced by either dbDNA^TM^ or plasmid template showed similar transgene expression over 30 days. Despite demonstrating similar efficacy when titre matched, overall titres for dbDNA^TM^ derived LVV were noticeably lower than for plasmid, indicating the need to optimise the system for dbDNA^TM^ to achieve industry-leading titres.

In this study, we demonstrate that dbDNA^TM^ can be optimised for the manufacture of high titre LVV. This work was carried out in the context of the more widely used and clinically relevant third-generation quadruple transfection production system [18], using suspension cell culture for enhanced scalability. Here, we identified optimised transfection conditions for dbDNA^TM^ that led to the rescue of total viral particle titres. However, these conditions did not improve infectious titres of dbDNA^TM^-derived LVV, which remained 2 logs lower in comparison to plasmid-derived LVV. We subsequently identified a possible mechanism to explain the observed low packaging efficiency of viral genomic RNA (vgRNA) derived from dbDNA^TM^ transfer vectors, whereby inefficient 3’ end processing could be leading to degradation of the 3’ LTR. Finally, we used vector engineering to overcome inefficient 3’ end processing and ultimately demonstrate that dbDNA^TM^ could be used to produce a highly clinically relevant CAR19h28z LVV with infectious titres equivalent to that achieved using plasmid-based methods.

## Results

### Comparison of dbDNA^TM^ and plasmid as templates for lentiviral vector production

**Fig. 1:**
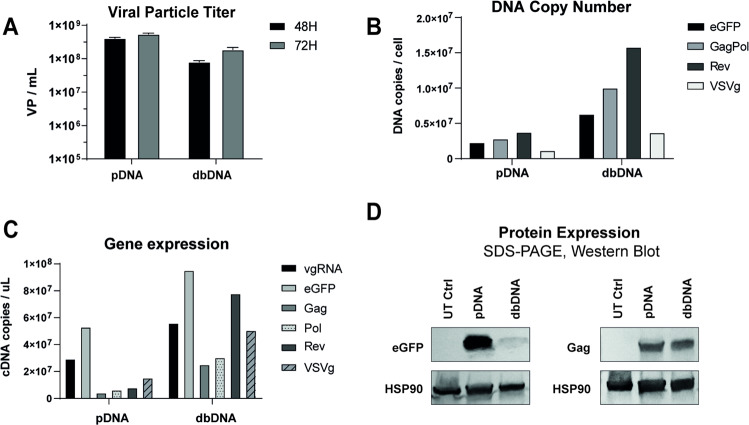
Characterisation of dbDNA transfection and expression. DNA copy number, gene expression, and protein levels were analysed in Viral Production Cells (VPC) transfected to produce lentiviral vector (LVV). **A** Total particle titre in supernatants from cells transfected with 1 μg/mL total DNA and a construct mass ratio of 2:1:1:1, measured by p24 ELISA 72 h post transfection. Transfections were performed in duplicate in 50 mL cell culture shake flasks. Error bars represent the standard deviation between replicates. **B** DNA vector copies per cell measured by qPCR. **C** Transcript abundance measured by RT-qPCR. Probe vgRNA was used to quantify full-length viral genomic RNA generated from the 5’ LTR, and eGFP was used for total RNA transcribed from the transfer vector. **D** Western blots showing eGFP and Gag protein levels in producer cell lysates, demonstrating low protein in dbDNA transfected cells relative to transcript abundance.

Linear, close-ended dbDNA^TM^ and circular plasmid are structurally different necessitating optimisation of transfection conditions for dbDNA^TM^ to ensure similar transfection efficiencies to plasmid. To enable a relevant comparison of the two topologies for vector production, we first optimised conditions for dbDNA^TM^ transfection in Viral Production Cells (VPC), a HEK293F-derived suspension cell line optimised for LVV scale-up, using a standard CMV-eGFP reporter (Supplementary Fig. S1A). We performed a side-by-side comparison of dbDNA^TM^ and plasmid as templates for LVV production in the third-generation transient transfection system. VPC were transfected with 4 DNA constructs encoding an eGFP transfer vector, GagPol, Rev, and VSVg at a standard construct mass ratio of 2:1:1:1, using 1 μg/mL of total DNA and DNA:PEI ratio of 1:2 for plasmid and 1:3 for linear dbDNA^TM^. Supernatant was harvested at 48 and 72 h post transfection, and total particle vector titres were determined by p24 ELISA. Despite having similar transfection efficiencies (Supplementary Fig. S1B), total viral particles titres (VP/mL) using dbDNA^TM^ were 5–10-fold lower than using plasmid (Fig. 1A), suggesting further optimisation was required to maximise yields from dbDNA^TM^ templates.

We hypothesised that the unique structure of dbDNA^TM^ might lead to differences in transfection and expression in producer cells, thereby impacting LVV titres. To explore this, RT-qPCR was used to analyse DNA copy number as well as transcript abundance in producer cells at 72 h post transfection. This revealed that cells transfected with dbDNA^TM^ contained 3–4-fold more DNA copies per cell of each construct compared to plasmid transfection (Fig. 1B). Equally, transcript abundance was 2–10-fold higher (Fig. 1C), suggesting that dbDNA^TM^ displayed a unique expression profile. These observations could not be fully explained by the smaller size of the dbDNA^TM^ vectors, which gives an average copy advantage relative to plasmid of only 1.5× when mass matched. Normalising transcript abundance to copy number further revealed that, despite similar or greater transcription efficiency of dbDNA^TM^ accessory constructs in comparison to plasmid, the dbDNA^TM^ transfer vector produced half the transcripts per DNA molecule as its plasmid equivalent (Supplementary Fig. S1C). Interestingly, higher transcript abundance did not translate to higher protein expression. Despite a 2-fold excess of *eGFP* transcripts in dbDNA^TM^ transfected cells, eGFP protein levels were greatly reduced compared to plasmid. Equally, Gag protein levels were similar for both plasmid and dbDNA^TM^ despite a 6-fold increase in the abundance of transcripts in dbDNA^TM^ transfected cells (Fig. 1D). These differences in DNA copy number and transcript abundance further correlated with a decrease in the rate of cell proliferation (data not shown). Collectively, these data suggested that the unique transfection and expression profile of dbDNA^TM^ could be overloading transfected cells during LVV production, leading to cellular stress, translational shutdown, and, thus, lower vector titres.

### Decreasing dbDNA^TM^ input and optimising construct ratios leads to high total particle titres

**Fig. 2:**
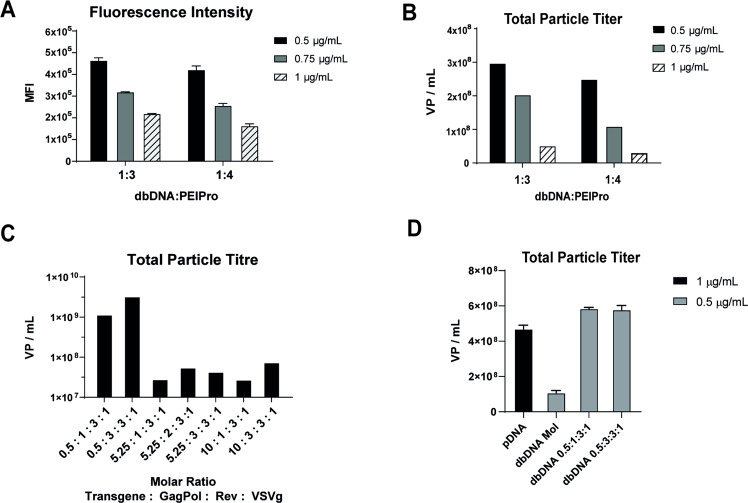
Optimisation of DNA input and construct ratios rescues total particle titres. **A** Mean fluorescence intensity of eGFP and **B** total viral particle titre (VP/mL) of LVV produced in cells transfected with decreasing total input dbDNA^TM^ and the indicated ratios of DNA:PEI. **C** High-throughput optimisation of construct ratios was performed using 0.5 μg/mL total input dbDNA^TM^ in 15 mL bioreactors in an automated Ambr15 cell culture system (Sartorius). Total viral particle titres from a selection of conditions are shown to demonstrate rescue of titres with reduced transfer vector. **D** Total viral particle titre (VP/mL) of LVV produced using plasmid at a mass construct ratio of 2:1:1:1, or dbDNA^TM^ using the molar equivalent to plasmid (Mol), or the optimised molar construct ratios of 0.5:1:3:1 and 0.5:3:3:1.

The difference in dbDNA^TM^ expression profile indicated that transfection conditions and construct ratios required further optimisation to achieve titres on par with industry standards. Using the same construct ratio as above (2:1:1:1), we first assessed the effect of total DNA input by transfecting cells with 0.5, 0.75, or 1.0 μg/mL dbDNA^TM^. Samples were harvested at 72 h post transfection for analysis of transfection efficiency and total VP titre. We observed a dose-dependent increase in mean fluorescence intensity (MFI) upon reduction of total dbDNA^TM^ (Fig. 2A), which correlated with a 6-fold increase in VP titres in samples transfected with 0.5 µg/mL dbDNA^TM^ (Fig. 2B). Decreased dbDNA^TM^ input also correlated with an increase in cell proliferation (data not shown), supporting our hypothesis that decreasing cellular stress would result in improved protein output and higher viral particle titres.

We next performed design of experiments (DoE) analysis and high-throughput optimisation of construct ratios using the automated bioreactor Ambr15 system. It is common practice to use mass-based construct ratios in LVV production. However, this has the disadvantage of leading to changes in construct copy numbers transfected when using different expression cassettes, which has important implications for the translation of transfection conditions. For this reason, we chose to perform construct ratio optimisation using molar ratios, where values represent the ratio of construct copy number and not mass. A preliminary small plate screen previously identified optimal ratios of 3 and 1 for Rev and VSVg (data not shown), thus subsequent efforts were focused on the eGFP transfer vector and GagPol. 0.5 μg/mL total input dbDNA^TM^, ratios of 0.5–10 and 1–3 were explored for eGFP and GagPol, respectively, whilst maintaining Rev and VSVg fixed at 3 and 1 (data not shown). Total VP titres were analysed 72 h post transfection. Trend analysis revealed that VP titres were significantly improved with reduced eGFP transfer vector and increased GagPol (example conditions are shown in Fig. 2C). The highest titre of 3.25 × 10^9^ VP/mL was achieved using the construct ratio of 0.5:3:3:1, reaching levels on par with industry standards. To confirm these results, a comparability study was performed between the standard plasmid condition (2:1:1:1; 1 μg/mL) and dbDNA^TM^ (0.5 μg/mL), using both the molar equivalent to plasmid and the two optimised ratio conditions of 0.5:1:3:1 and 0.5:3:3:1. Use of both optimised dbDNA^TM^ ratio conditions confirmed that total VP titres were reproducibly equivalent to plasmid (Fig. 2D)

### Increasing the transfer vector ratio does not improve infectious particle titres

**Fig. 3:**
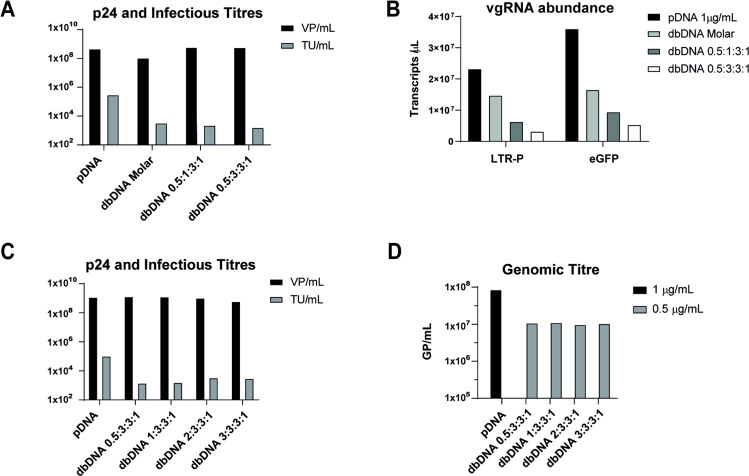
Increasing transfer vector has little effect on low infectious and genomic titres. **A** Infectious titres (TU/mL) of LVV generated in Fig. 2D, measured by flow cytometry of transduced VPCs 48 h post transduction. **B** Viral genomic RNA (vgRNA) abundance in producer cells from panel (**A**), measured by RT-qPCR using probes against LTR-P and eGFP. **C** Viral particle (VP/mL) and infectious titres (TU/mL) of LVV generated using 1 μg/mL plasmid at a mass ratio of 2:1:1:1, or 0.5 μg/mL dbDNA at the indicated molar ratios. **D** Genome-containing particle titre (GP/mL) of LVV from panel **C** measured by RT-qPCR.

Having identified conditions that yielded high particle titres, we next aimed to evaluate the infectivity of dbDNA^TM^-derived particles. To address this, we transduced HEK293T cells with supernatants harvested from producer cells (Fig. 2D). Transducing units per mL (TU/mL) were quantified at 48 h post transduction by flow cytometry. This revealed that dbDNA^TM^ derived particle infectivity was approximately 100-fold lower in comparison to plasmid. Furthermore, decreased infectious titres correlated with a low abundance of lentiviral genomic RNA (vgRNA) (Fig. 3B), suggesting that the observed decrease in infectivity could be caused by the presence of empty LVV particles due to insufficient transfer vector. Surprisingly, increasing the ratio of eGFP transfer vector failed to significantly improve infectious titre (Fig. 3C) despite leading to a clear dose-dependent increase in both vgRNA and MFI in producer cells (Supplementary Fig. S2A, B). Furthermore, RT-qPCR analysis of viral particles confirmed that the increase in vgRNA abundance did not result in increased genome-containing particles (Fig. 3D). Taken together, these data suggested that vgRNA derived from the standard dbDNA^TM^ LV-eGFP transfer vector was not being efficiently incorporated in lentiviral particles.

To address the 2-fold decreased abundance of vgRNA in our dbDNA^TM^ system (Supplementary Fig. S2A), we evaluated the effect of increasing the eGFP transfer vector up to 10-fold. In parallel, VSVg was modestly increased, as VSVg transcripts were also found to be low relative to plasmid conditions by RT-qPCR (data not shown). While a small improvement was seen using a ratio of 4:3:3:4, this was not significant (Supplementary Fig. S3A, B). Nevertheless, subsequent productions using dbDNA^TM^ were performed using the ratio of 4:3:3:4. Collectively, these data suggested the possibility of a fundamental flaw with the architecture of the dbDNA^TM^ transfer vector, whereby resulting vgRNA transcripts were not effectively being packaged.

### Engineering of transfer vector architecture greatly improves infectivity

To address the packaging defect in the transfer vector, we sought to determine an underlying mechanism. We posited that the weak poly(A) activity intrinsic to SIN-LV [19, 20] vectors combined with the lack of downstream template in dbDNA^TM^ could disrupt transcription termination through premature drop-off of RNA polymerase II (RNA pol II) from the template, or RNA pol II looping around the 3’ closed end of dbDNA and initiating anti-sense transcription, leading to unstable vgRNA transcripts prone to degradation. RNA-seq of the LV-eGFP transfer vector revealed a significant drop-off in read abundance across the 3’ LTR in dbDNA^TM^ relative to plasmid (Supplementary Fig. S4). This was associated with detectable anti-sense transcription across the 3’ stuffer in dbDNA^TM^, possibly indicating a further degree of interference.

**Fig. 4:**
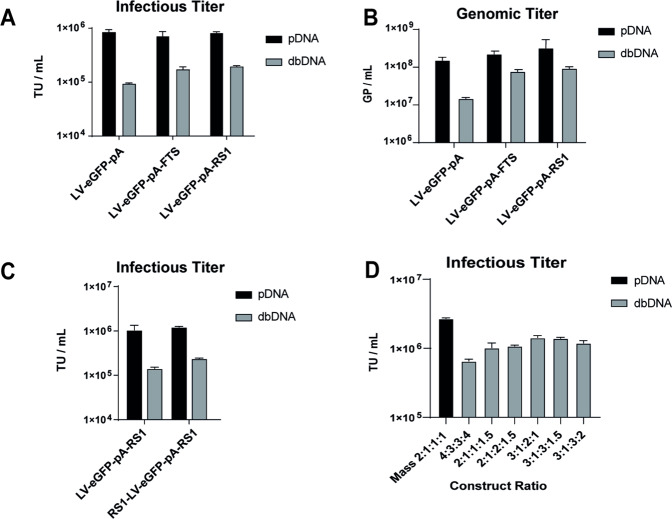
Engineered transfer vector architectures partially rescue LVV infectivity. **A** Infectious (TU/mL) and (**B**) genome-containing particle (GP/mL) titres of LVV produced using 1 μg/mL plasmid (mass 2:1:1:1) or 0.5 μg/mL dbDNA (molar 4:3:3:4) and the indicated transfer vectors. **C** Infectious titre of LVV produced with as described in panel (**A**) using LV-eGFP-pA-RS1 and LV-RS1- eGFP-pA-RS1 transfer vectors. **D** Infectious titre of LVV produced using 0.7 μg/mL dbDNA and the indicated molar construct ratios. Error bars represent the standard deviation between replicates.

To address the drop off in read abundance, we engineered a transfer vector with a strong late SV40 poly(A) immediately downstream of the 3’ LTR and upstream of the 250 bp stuffer region (LV-eGFP-pA) (Supplementary Fig. S5). RNA-seq analysis confirmed an improvement in coverage across the 3’ LTR in this vector; however, anti-sense transcription was still detected through the poly(A) (Supplementary Fig. S6A). Furthermore, LV-eGFP-pA yielded a 10-fold improvement in infectious titres of LVV particles, from 100-fold lower to 9-fold lower compared to the plasmid control (Fig. 4A). To further improve processing and therefore titres, an 806-nt sequence derived from the mouse β-globin gene termed the F region, previously shown to exhibit strong termination activity [21], was inserted downstream of the poly(A) (LV-eGFP-pA-FTS). As a control, a third vector was engineered with an 806-nt random stuffer sequence in place of the F region (LV-eGFP-pA-RS1), giving both vectors a total of 1 kb of template downstream of the SV40 poly(A) (Supplementary Fig. S5). Both vectors led to a further 2.5-fold improvement in infectious titres in a sequence-independent manner, indicating that the presence of template rather than specific termination signals was key. Importantly, both total particle titres and overall vgRNA abundance were similar for the three different vectors (Supplementary Fig. S7A, B). However, genomic titres were improved in the presence of the LV-eGFP-pA-FTS and LV-eGFP-pA-RS1 (Fig. 4B), strongly supporting the hypothesis that the increase in infectious titres was a result of improved processing and packaging and not due to higher transcription. RNA-seq performed on LV-eGFP-pA-RS1 further confirmed that the addition of the 3’ 1 kb random spacer completely abrogated anti-sense transcription at the 3’ end of dbDNA^TM^ (Supplementary Fig. S6B).

An interesting observation from these studies was that cells transfected with the new dbDNA^TM^ transfer vectors proliferated more rapidly than those transfected with dbDNA-LV-eGFP. It’s possible that small dsRNA fragments, generated by the looping of RNA pol II around the end of dbDNA-LV-eGFP, could have deleterious effects on cells by activating the RNA-induced silencing complex (RISC), a multiprotein complex that binds single-stranded RNA fragments such as microRNA or double-stranded small interfering RNA to mediate gene silencing. We suggested that this would not occur with the optimised transfer vectors, where anti-sense transcription was resolved. We therefore asked whether titres might be further improved, using these engineered architectures, by increasing total DNA input. Using LV-eGFP-pA-RS1, we performed productions in which we incrementally increased total input DNA from 0.5 to 1 µg/mL (Supplementary Fig. S8A, B). As expected, cell counts progressively declined with increasing DNA concentration. Interestingly, peak titres were achieved using 0.7 µg/mL, a condition in which cell growth was equivalent to that achieved using the plasmid control. Based on these data, we concluded that a total input of 0.7 µg/mL was optimal for subsequent LVV productions.

We next asked whether dbDNA^TM^ transfer vectors also required additional template upstream of the 5’ CMV/LTR, to allow space for RNA polymerase II to bind, track, and efficiently initiate transcription of the full-length vgRNA. To test this, a 1 kb random spacer sequence was inserted upstream of the CMV/5’ LTR in addition to the 3’ SV40 poly(A) and 3’ spacer (LV-RS1-eGFP-pA-RS1). While no change in expression profile was detected by RNA-seq (data not shown), we observed that the addition of a 5’ spacer sequence improved infectious titres by 2-fold (Fig. 4C). We hypothesised that the addition of the upstream spacer could increase the amount of vgRNA available for packaging through improvements to overall transcription levels without affecting normalised transcription profiles. Having determined differences between the expression profiles of LV-RS1-eGFP and the initial LV-eGFP transfer vector, we performed high-throughput optimisation of construct ratios using the optimised DNA input of 0.7 µg/mL and the final transfer vector architecture (data not shown). We observed that several conditions in which transfer vector and Rev were increased yielded significant improvements in infectious titres compared to the previous dbDNA^TM^ condition of 4:3:3:4. In particular, titres of up to 1.4 × 10^6^ TU/mL were achieved using the ratio of 3:1:2:1 (Fig. 4D). This was an improvement relative to Fig. 3B of over 3 orders of magnitude.

### Addition of spacers to accessory constructs leads to equivalent infectious titres for plasmid and dbDNA^TM^ CAR-T lentiviral vector

**Fig. 5:**
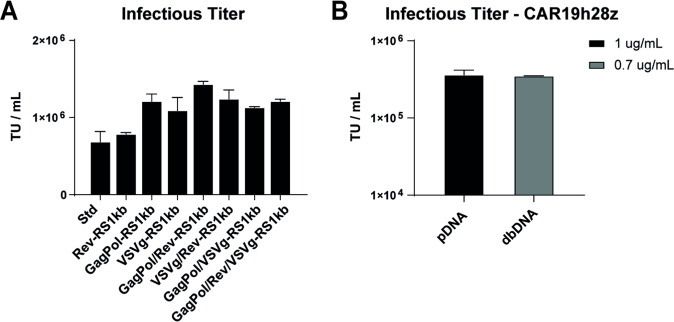
Evaluation of 3’ RS1kb in accessory constructs leads to rescue of dbDNA-derived LVV. **A** Infectious titre of LVV produced using 0.7 μg/mL dbDNA at a molar construct ratio of 4:1:2:1. The LV-RS1-eGFP-pA-RS1 transfer vector was used in combination with our standard accessory constructs (Std), and each accessory construct was iteratively swapped for the equivalent construct containing a 3’ RS1 element such that each construct was tested independently and in combination with all others. **B** Infectious titre of LVV produced using a CAR19h28z transfer vector, GagPol-RS1, Rev-RS1, and VSVg (0.7 μg/mL dbDNA at a molar ratio of 4:1:2:1). Error bars represent the standard deviation between replicates.

The improvement in LVV titres achieved through optimising the dbDNA^TM^ vector architectures and transfection ratios led us to ask whether titres could be further improved by adding 3’ spacers to the accessory constructs. Here, 5’ spacers were not included to keep additional template to a minimum. Given the 3’ spacer demonstrated the greatest improvement in the context of the transfer vector, we hypothesised that the addition of this region to the accessory constructs would be sufficient to further improve titres. Productions were carried out in which each accessory construct was swapped for the equivalent accessory +3’ RS1kb, individually or in groups, such that all combinations were evaluated (Fig. 5A). While addition of RS1kb to Rev did not yield any improvement in infectious titre, addition of RS1kb to GagPol or VSVg substantially improved infectivity. Interestingly, while Rev-RS1kb alone did not improve titres, the highest titres were achieved when GagPol-RS1kb and Rev-RS1kb were combined.

Finally, we sought to show that the demonstrated improvements in LVV yield from dbDNA^TM^ would translate to a therapeutically relevant lentiviral payload. As a model system, we used a publicly available sequence for an anti-CD19 CAR (CAR19h28z) [22] with demonstrated efficacy in a clinical trial [23]. We generated a LVV expressing CAR19h28z from our optimised dbDNA^TM^ architecture, including the downstream SV40 LpA and flanking RS1kb (LV-RS1kb-1928z-LpA-RS1kb). VPC were co-transfected with LV-RS1kb-1928z-LpA-RS1kb, GagPol-RS1kb, Rev-RS1kb, and VSVg using the optimised molar ratio of 4:1:2:1 for dbDNA (0.7 μg/mL DNA; 1:3 DNA:PEI), and a mass ratio of 2:1:1:1 for plasmid (1 μg/mL DNA; 1:2 DNA:PEI). Supernatants were harvested at 72 h post transfection, and infectious titres were quantified in THP-1 cells by CD19 FACS. The molar ratio of 4:1:2:1 was used for dbDNA^TM^ instead of 3:1:2:1 as further titration experiments performed using the final set of optimised constructs demonstrated that this increase in transfer vector further improved infectious titres (data not shown). We observed that use of fully optimised constructs and transfection conditions resulted in equivalent infectious titres between dbDNA^TM^ and pDNA for LVV CAR1928z (Fig. 5B), demonstrating that dbDNA^TM^ can be used as a rapid and effective alternative to plasmid for the manufacturing of high titre, clinically relevant LVV.

## Discussion

In this study, we have demonstrated that dbDNA^TM^ can be used as an efficient template for the manufacture of third-generation LVV. Using engineered vector architectures and optimised transfection conditions, we were able to produce a CAR19h28z LVV with equivalent infectious titres as achieved using plasmid. We have previously shown that dbDNA^TM^ could be used to make second-generation LVV particles that demonstrated similar infectivity to plasmid-derived LVV when titre matched; however, overall titres were significantly lower for dbDNA^TM^ than plasmid [12].

This observation became more challenging when we attempted to translate these findings to the more clinically relevant third-generation transfection system. Here, viral particle titres were consistently 5–10-fold lower than plasmid despite a substantial excess in vector copy number and gene expression in dbDNA^TM^ transfected producer cells. One possible explanation is that the smaller size and linear nature of dbDNA^TM^ have implications for the compaction and/or composition of PEI:DNA complexes [24], ultimately enabling more efficient entry of dbDNA^TM^ into cells. The increased productivity that we observed for each dbDNA^TM^ accessory construct relative to plasmid further suggests that a greater proportion of transfected dbDNA^TM^ reaches the nucleus to be transcribed, which in other therapeutic contexts would certainly present a large advantage. Ultimately, this enabled the production of equivalent LVV titres using a fraction of the dbDNA^TM^ input material that is required for plasmid. Despite achieving equivalent VP titres through transfection optimisation, infectivity of dbDNA^TM^ derived VPs remained 3 orders of magnitude lower than plasmid when using the standard LV-eGFP transfer vector. Increasing the abundance of vgRNA more than 6-fold had no impact on infectivity or genomic titre. This suggested an incompatibility between the linear dbDNA^TM^ structure and the basic LVV transfer vector architecture, whereby vgRNA was not efficiently processed during transcription and resulting transcripts were incompatible with packaging.

Genome-wide ChIP-seq analysis of eukaryotic genomes has shown that transcription of genes which undergo poly(A) addition is associated with RNA pol II pausing between 1 and 6 kb past the site of the poly(A) signal [21, 25–28]. It is thought that this pausing is essential for efficient 3’ end processing of nascent transcripts through interactions between processing factors bound to the C-terminal domain of RNA pol II and the poly(A) signal [29–31]. The distance at which RNA pol II pauses has further been shown to be dependent upon the strength of the associated poly(A) [32] that may reflect the slower rate at which weak poly(A) signals recruit factors necessary for processing [33]. Furthermore, there are numerous examples of poly(A) signals for which flanking RNA is essential for full activity, either through recruitment of additional *trans-*acting factors or through direct interactions between flanking RNA and processing factors at the poly(A) [34–40], with the emerging picture being of a core poly(A) signal within a larger poly(A) domain.

It is also well documented that self-inactivating LVV (SIN-LV) suffer from leaky transcriptional termination due to the deletion of the U3 region of the 3’ LTR [19, 20], believed to contain termination enhancer motifs necessary to support efficient 3’ end processing of nascent transcripts [41, 42]. It is therefore possible that SIN-LV vectors could require significant template downstream of the weak 3’ LTR poly(A) to enable RNA pol II pausing and efficient transcript processing. This template is not present in linear dbDNA^TM^, possibly leading to premature drop-off of RNA pol II from the template, or alternatively looping of RNA pol II around the TelRL resulting in anti-sense transcription. Addition of a strong termination signal downstream of the 3’ LTR dramatically improved packaging and infectivity of dbDNA^TM^ -derived LVV, and this correlated with increased read abundance across the 3’ LTR. Antisense transcription was still detected through the spacer and the termination signal, however, potentially due to insufficient template downstream from the termination signal. It is, therefore, possible that a proportion of transcripts could have disrupted 3’ processing through self-annealing across the poly(A) region. In agreement with this, extending the spacer to 1 kb completely abrogated anti-sense transcription and led to a further improvement in packaging and infectivity.

Enhancing 3’ end processing of transcripts is particularly important in the context of SIN-LVV manufacturing, as demonstrated by the improvement in infectious titre observed for both plasmid and dbDNA^TM^ with the addition of the SV40 poly(A) downstream of the 3’ LTR. Addition of the 3’ random spacer specifically improved dbDNA^TM^ titres, further demonstrating the requirement for additional template to support RNA pol II run-off and efficient transcript processing. This may also explain the improvement seen using dbDNA-Rev-RS1kb, which is also terminated by a comparatively weak HIV LTR poly(A). We have not observed any detectable improvements in expression with the addition of a 3’ spacer to standard dbDNA^TM^ expression vectors, however, which contain simple viral or eukaryotic driving elements and strong termination signals as a rule, suggesting this phenomenon is specific to the context of LVV constructs. Indeed, dbDNA^TM^ has successfully been shown to serve as a complete replacement for plasmid in the robust and scalable manufacturing of AAV without the requirement for any vector engineering [11].

LVVs are the preferred vector for many applications due to their large packaging capacity, ability to transduce most cell types, and stable expression in dividing tissues. Despite this, major obstacles around scalability, cost-effectiveness, and regulatory compliance remain in the development and advancement of LVV-based therapies. The intrinsic fragility of enveloped viruses makes large-scale manufacturing of LVV very challenging from a bioprocessing perspective, and so generating sufficiently high titres to address diseases with large or underprivileged patient populations in a cost-effective manner remains a significant challenge. Considering that ex vivo applications for HSC gene therapies require doses in the range of 1–500 × 10^9^ TU per patient [43], the urgent need for improved scalability becomes self-evident.

A major hurdle in scaling up LVV production lies in the sourcing of large quantities of GMP grade plasmid, which leads to long lead times and drives up operational costs due to the limited supply chain and cost of plasmid manufacturing [43, 44]. Enzymatically amplified linear dbDNA^TM^ may address this challenge in several ways. The speed and scalability with which GMP material can be manufactured reduces lead times from up to 9 months for plasmid [45] to approximately 50 days for dbDNA^TM^, enabling rapid and agile discovery programs and industrial-scale manufacturing of clinical candidates. The cell-free amplification process additionally removes the possibility of sequence instability during bacterial propagation, which can reduce plasmid yields by compromising sequence integrity of the final product. While the unit cost of dbDNA^TM^ at a small scale is comparatively greater than the equivalent amount of plasmid, the enhanced speed and consistency of manufacturing combined with a reduced requirement for starting material may ultimately lead to improved cost-effectiveness. This effect is only enhanced at larger scales of production, where the cost of manufacturing progressively decreases relative to plasmid. In the context of large-scale GMP plasmid production, costs related to infrastructure and personnel are substantially increased leading to high operational costs. In contrast, the simplicity of the dbDNA^TM^ manufacturing platform means that the predominant costs associated with large-scale GMP production are the cost of goods related to production enzymes, resulting in significant scale economies.

Given its advantages from a manufacturing perspective, we set out to demonstrate that dbDNA^TM^ could be used as an alternative to plasmid for LVV manufacturing, thus providing a possible solution to the plasmid bottleneck. Using an optimised suite of vectors and transfection conditions, we ultimately showed that dbDNA^TM^ could be used to produce infectious titres of the clinically relevant CD19h28z LVV equivalent to those obtained using plasmid-based methods. Moreover, this was performed using 70% of the DNA input required for plasmid. While the potential of dbDNA^TM^ is demonstrated here, its use for LVV manufacturing remains a new development and has thus far only been tested in limited production contexts and with limited transfer vector sequences. Therefore, researchers seeking to adopt dbDNA^TM^ technology will face the challenge of optimising conditions within their own LVV production platforms and, in the first instance, should perform head-to-head comparisons with plasmids. Particularly when transferring to adherent production cell systems or when using alternative transfection reagents, total DNA input and the ratio of transfection reagent to DNA will need to be evaluated, as the optimal conditions for plasmid and dbDNA^TM^ might differ.

Considerable work was carried out here to achieve parity with plasmid. However, this ultimately came from a mechanistic difference between plasmid and dbDNA^TM^ in the processing of SIN-LV RNA genomes, which was resolved through the addition of a strong termination signal and spacer sequences to the dbDNA^TM^ transfer vector. These elements lie fully outside of the viral LTRs and thus should have no impact on any desired transfer vector sequence. It is, therefore, likely that further development of production parameters for new vector sequences will be limited to standard optimisation of construct ratios. Indeed, the conditions established for our eGFP payload translated directly to a CAR-T payload. It is also important to note that titres obtained here were not generated in a controlled environment and to the usual industry standards, and therefore we cannot discard the possibility that there will be challenges when translating this system to large-scale LVV manufacturing. Here, we focused on developing constructs and conditions that would yield equivalent titres using dbDNA^TM^ as obtained with plasmid-based methods, with the understanding that higher overall titres will be achieved through optimisation and adaptation to scaled-up industry manufacturing platforms.

For companies seeking to transition in the context of a clinical-stage candidate, additional considerations will be required. Transition from plasmid to dbDNA^TM^ can be done during development or at the life cycle maintenance stage of a marketed product. The regulatory acceptability of such transition will be based on the establishment of comparability through a combination of analytical testing, biological assays, and, in some cases, non-clinical and clinical data to demonstrate that the change has no negative impact on quality, safety and efficacy of the final product. If a manufacturer can provide assurance of comparability through analytical studies alone, non-clinical or clinical studies with the post-change product are not warranted. It’s worth noting that Chemistry, Manufacturing and Controls of dbDNA^TM^ has gone through the scrutiny of regulatory bodies such as FDA and MHRA for its utility in various gene therapy applications under development.

In summary, we show here that dbDNA^TM^ can be used as an alternative solution for LVV manufacturing. The use of dbDNA^TM^ offers benefits from both a regulatory and a manufacturing standpoint due to the elimination of bacterial propagation sequences, the significantly shorter lead times for multi-gram scale GMP material, and a reduced requirement for input material, which will help to further reduce the material costs of LVV therapies. Another key advantage of dbDNA^TM^ lies in its ability to amplify sequences that are difficult to propagate in bacteria, of which a number have been identified to be therapeutically relevant [46, 47]. There is equally no theoretical limit to the size of dbDNA^TM^ vectors, with at least 20 kb constructs routinely being manufactured. While this may not be relevant to LVV therapies, it is becoming increasingly clear that the use of long and complex genomic sequences is critical to achieving robust and stable transgene expression in certain tissues, and so this may provide a key benefit for non-viral gene therapy applications. The use of dbDNA^TM^ may thus enable the therapeutic development of gene therapy strategies for the treatment of previously intractable genetic diseases.

## Methods

### pDNA/dbDNA design, cloning and manufacture

All sequences for the standard DNA lentiviral constructs (eGFP transgene, GagPol, Rev and VSG) were obtained from Addgene (www.addgene.org) choosing from widely used lentiviral third-generation production systems. Those sequences were synthesised de novo and cloned into Touchlight’s proTLx backbone at Genewiz (www.genewiz.com) or Genscript (www.genscript.com). The resulting plasmids (pDNA) were then used as templates to generate the equivalent dbDNA versions through Touchlight’s dbDNA^TM^ manufacturing process [48].

All modifications to the standard eGFP transgene to include the new elements described in this paper were also synthesised de novo, by either Genewiz or Genscript, and underwent the same procedure for pDNA and dbDNA^TM^ manufacturing at Touchlight Ltd. The CAR-T gene was designed based on the 1928z sequence described by the Sadelain lab in Eyquem et al. [49]

### Western blotting

Cells were harvested, resuspended in laemmli buffer (Bio-Rad, 1610747) plus beta-mercaptoethanol (Sigma, M3148) and boiled at 100 °C for 10 min. Samples were loaded into 10% SDS-PAGE gel (Bio-Rad, 4561031) and run at 50 v for 30 min followed by 100 v (Bio-Rad, power pack basic) in 1× running buffer (Bio-Rad, 1610732). Transfer was performed using the iBlot2 dry blotting system. Following transfer, nitrocellulose membranes (Invitrogen, IB23001) were blocked in 5% BSA in 1 × TBS (Bio-Rad, 1706435) for 30 min. Membranes were incubated with antibodies against HSP90 (CST, 4877S) p24 (Abcam, ab9071) or eGFP (CST, 2955S) according to the manufacturer’s instructions. Membranes were washed (TBS + 0.1% Tween-20) and incubated with secondary antibodies (anti-mouse, Amersham, NXA931 or anti-rabbit, Amersham, NA934) according to manufacturer instructions, for 1 h at room temperature. Membranes were imaged using iBright1500 (Invitrogen) and Pierce ECL blotting substrate (Thermo, 32132).

### Cells and cell culture

HEK293F cells (Gibco Viral Production Cells, A35347) were cultured following the manufacturer’s recommendations, either in Erlenmeyer flasks with vent cap using LV-MAX Production Media (Gibco, A3583401) at 50 mL culture volume in a platform shaking incubator at 37 °C, 8% CO_2_ and 125 rpm or in an automated 48-bioreactor Ambr15 system (Sartorius) at 10 mL culture volume and 400 rpm. Cell density was quantified using the countess^TM^ automated cell counter (Invitrogen) by trypan blue exclusion (Gibco, 15250061) and cultures were maintained between 0.5 and 6 × 10^6^ cells/mL. Cells were used up to passage number 12. HEK293T cells (Takara, 632180) were cultured in DMEM (Gibco, 41965-039) supplemented with 10% FBS (Sigma, F4135) and 2 mM L-Glutamine (Sigma, G7513) and incubated at 37 °C in 5% CO_2_ (Hera Cell). Cells were detached with trypsin-EDTA (Gibco, 25200-072) and passaged three times per week, 1:4–1:10, to maintain sub-confluency. Cells were used up to passage number 8. THP-1 (ATCC, TIB-202) were cultured in RPMI (Gibco, 61870-036) supplemented with 10% FBS (Gibco, 10082-147) and incubated at 37 °C in 5% CO_2_ (Hera Cell). Cell density was quantified as per HEK293F cells. THP-1 were passaged 2 times per week and maintained between 2 and 8 × 10^5^ cells/mL. Cells were used up to passage number 12. Cells were not authenticated or tested for mycoplasma given the adherence to low passage numbers, after which cells would be discarded and a new batch thawed from a fresh viral direct from the manufacturer.

### Lentiviral production: cell culture, transfection and harvest

The day before transfection, 50 mL cultures of HEK293F cells were established at 1 × 10^6^ cells/mL. On transfection day, a total of 1–0.5 μg/mL of pDNA or dbDNA^TM^—containing a mass/molar ratio of the four lentiviral constructs (eGFP transgene, GagPol, Rev and VSVg)—was transfected using PEIPro (PolyPlus Transfection) as transfection reagent following manufacturer’s recommendations for suspension cells. Harvest was performed at 48 h/72 h post transfection by filtering the supernatants (0.45 μm) after centrifugation of the 50 mL cultures for 5 min at 1300 rpm. Supernatants were subsequently aliquoted and stored at −80 °C for later analysis. Cell pellets were resuspended and washed with 50 mL PBS (Sigma Aldrich, D8537) and then used for assessing cell density (Trypan Blue staining, Countess, Invitrogen), analysis of cellular eGFP expression using CytoFlex Flow Cytometer (Beckman Coulter) and cell pellets stored at −80 °C for later gene expression analysis.

### DNA delivery and gene expression analysis

From the packaging cell pellets, total DNA and RNA were extracted using DNeasy Blood and Tissue and RNeasy Plus Mini kits respectively from Qiagen (www.qiagen.com) following the recommended protocols. For DNA delivery, extracted total DNA was then analysed by singleplex qPCR analysis with a StepOnePlus qPCR (Applied Biosystems) using, in separate reactions, a custom TaqMan primers/probe set (IDT Technologies) against the lentiviral target sequence, together with a copy number standard curve using the adequate reference material, and the RNAseP TaqMan Copy Number Reference Assay (Applied Biosystems) together with a wild-type HEK293F genomic DNA standard curve to assess the number of DNA vector copies delivered per cell during transfection. For gene expression analysis, 1 μg RNA was used to synthesise cDNA with SuperScript III First-Strand Synthesis SuperMix for qRT-PCR (Thermo Fisher Scientific). cDNA was then analysed by duplex qPCR analysis using a custom FAM-dye TaqMan primers/probe set (IDT Technologies, https://eu.idtdna.com) against the lentiviral target sequence and a gene expression housekeeping gene, either GAPDH or 18S rRNA VIC-dye endogenous control (Applied Biosystems) together with a copy number standard curve using the adequate reference material to assess the normalised number of transcripts being generated. Prior to these assays, several TaqMan Primers/Probe sets for each target, except for eGFP, were designed using IDT’s PrimerQuest online tool (www.idtdna/primerquest) and then tested to select the best performing ones which sequences are described in Supplementary Table S1. For eGFP, we used a validated TaqMan gene expression assay (FAM) from Applied Biosystems (4331182, Assay ID Mr04097229_mr).

### Lentiviral samples analysis: total titre, infectious titre and genomic titre

For assessing total titres (viral particles per mL, VP/mL) from diluted lentiviral supernatants, a Lentivirus-Associated p24 ELISA Kit (Cell Biolabs, VPK-107-5) was used following the instructions provided by the manufacturer.

For measuring the infectious titre of eGFP LVV (Transduction Units per mL, TU/mL), adherent HEK293T (Lenti-XTM 293T, Takara, 632180) were cultured and seeded the day before infection in 6- or 24-well plates. The following day, cells were infected with serial dilutions of the lentiviral supernatants containing 8 μg/mL of Polybrene (Santa Cruz, sc-134220). Plates were centrifuged at 900 × g for 30 min at room temperature and then incubated for 72 h at 37 °C and 5% CO_2_. Then, 72 h post infection, cells were trypsinised, washed with PBS, and eGFP expression was analysed by Cytoflex Flow Cytometer (Beckman Coulter). Supernatant dilutions giving 5–25% of eGFP positive cells were used to calculate infectious titre (TU/mL) using the following formula: TU/mL = (*F* × *C*/*V*) × *D*, where *F* = frequency of GFP^+^ cells (%GFP^+^ cells/100), *C* = cell number per well seeded for transduction, *V* = volume of inoculum in mL (0.1 mL) and *D* = lentivirus dilution factor.

For measuring infectious titre of CAR19hCD28z LVV, 5 × 10^5^ THP-1 cells (ATCC, TIB-202) were seeded per well of a 24-well plate on the day of infection. Cells were infected with serial dilutions of LVV supernatants in a medium containing 8 μg/mL polybrene and centrifuged at 1000 × g for 1 h at RT. Then, 48 h after infection, cells were washed and stained with anti-mouse F(ab’)2 fragment IgG conjugated with Alexa Fluor 647 (115-606-003-JIR, Stratech) and analysed by FACS, as above, to determine CAR19h28z expression. Infectious titre was calculated as described above.

Genomic titre (genome particles per mL, GP/mL) was calculated using Takara’s Lenti-X qRT-PCR Titration Kit (631235), which requires RNA genome extraction from the lentiviral supernatants followed by quantification of lentiviral genome copies by qRT-PCR.

### Analyses

Flow cytometry data were analysed using FlowJo v10 software. qRT-PCR and ELISA data were analysed using Microsoft Excel. Figures from analysed data sets were plotted using GraphPad Prism 9 software. Data bars represent mean and error bars represent mean ± standard deviation.

### RNA sequencing

#### Read quality control

We used fastp version 0.20.1 [50] to control and improve the sequence read quality for each supplied sample dataset before starting downstream analysis. General quality profiling was performed, followed by removal of low-quality reads, trimming of low-quality bases and adapter removal. The processed fastq files obtained from fastp were used for read alignment.

#### Read alignment

Before aligning the reads we constructed custom reference genomes by concatenating the supplied construct reference sequences with the human genome (GENCODE, GRCh38.p13). This was done to avoid potential artefacts that might arise due to interference from background transcription (transcription of non-construct templates). Once the references had been constructed, reads from each sample group were aligned to their corresponding reference using STAR version 2.7.5c [51]. Both reference indexing and alignment were performed with default settings. The aligned reads were then sorted and indexed using SAMtools version 1.12 [52] to enable quantification of sense and antisense transcription.

#### Quantification of sense and antisense transcription

A custom algorithm was developed for parsing the aligned reads and assigning them individually as either sense or antisense counts at each nucleotide position of a given reference sequence. The algorithm is described below:

For each *aligned sample dataset*:

 For each corresponding *construct reference sequence*:

  For each *nucleotide position*:

   For each *aligned read*:

Is the read first mate in pair?

Is the read reverse aligned?

Sense counts + 1

Is the read forward aligned?

Antisense counts + 1

Is the read second mate in pair?

Get first mate in pair

Is first mate reverse aligned?

Sense counts + 1

Is first mate forward aligned?

Antisense counts + 1

Applying this algorithm across all aligned sample data sets and corresponding construct reference sequences yielded sense and antisense counts per sample, reference, and nucleotide position. These data were then normalised and integrated to facilitate visualisation and comparison between sample groups. Data normalisation was done by dividing the sense and antisense counts at each nucleotide position by the total number of reads that aligned to the construct reference sequence in question. Data integration was then performed by calculating the average sense and antisense counts per construct reference sequence, of all samples per sample group. Maximum and minimum counts across samples were also recorded for each nucleotide position, to be used for visualising count variability.

#### Visualisation of sense and antisense transcription

Data visualisation was performed in R using ggplot2. A custom script was developed for iterating over each sample group and constructing reference, generating one plot per iteration. Each sample group/construct reference pair was plotted with normalised average and max-min counts along the y-axis and nucleotide position along the *x*-axis. Sense and antisense counts were split into separate series. Each plot was then annotated with template features.

## Supplementary information

Supplementary information

## Data Availability

Data generated and analysed during this study can be found within the published article and supplementary files, and additional data are available from the corresponding author upon reasonable request.
