# Supplementary material for: Enzymatically amplified linear dbDNATM as a rapid and scalable solution to industrial lentiviral vector manufacturing
Source: Gene Ther. 2022 May 24;30(1-2):122–31. doi: 10.1038/s41434-022-00343-4 (PMC9935383; doi:10.1038/s41434-022-00343-4)
Supplement: Supplementary file 1 — Supplementary information [file 41434_2022_343_MOESM1_ESM.pdf]

Supplementary Information

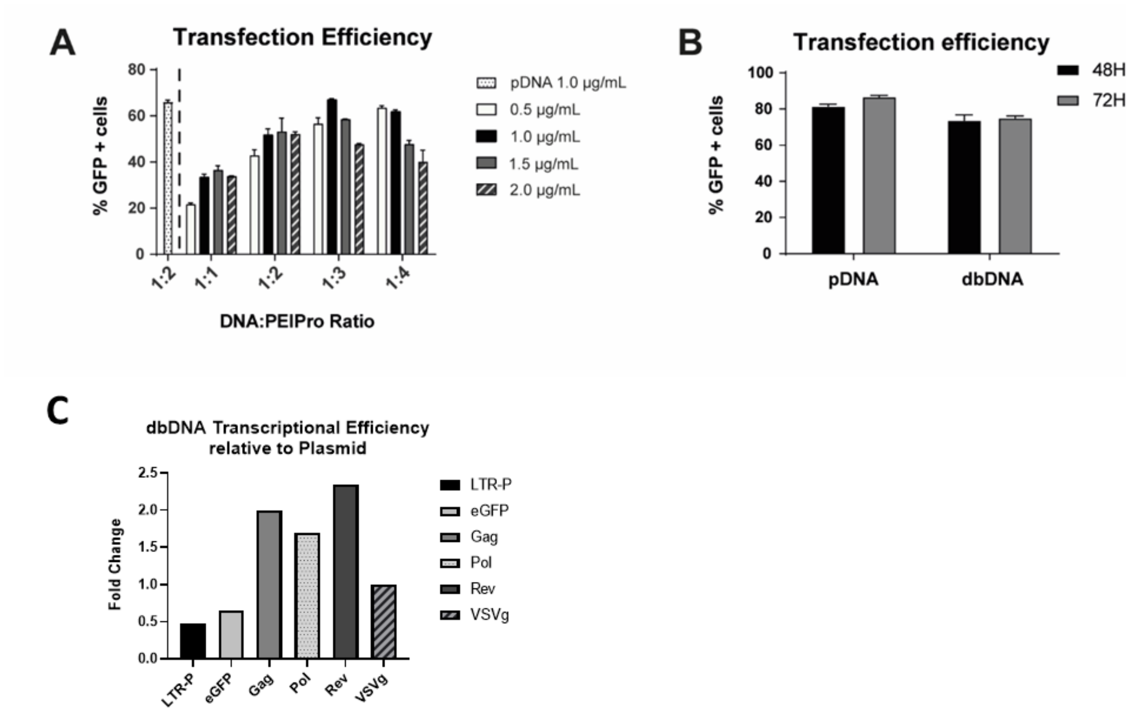

Figure S1. Optimisation of transfection conditions for dbDNA™

(a) 0.5 – 2.0 µg/mL of a dbDNA-eGFP reporter construct was transfected into suspension HEK293F cells reporter using a range of DNA:PEIpro ratios. 1 µg/mL pDNA-eGFP transfected using a DNA:PEIpro ratio of 1:2 was used as a control. Cells were analysed for transfection efficiency by flow cytometry 48 h post tranfection **(b)** HEK293F cells were transfected to produce lentiviral vector (LVV) using 1 ug/mL total pDNA or dbDNA and a construct ratio of 2:1:1:1 (Transgene: GagPol: Rev: VSVg). Transfection efficiency was measured by flow cytometry 72 h post transfection. Error bars represent the standard deviation between replicates **(c)** Transcriptional efficiency for each plasmid and dbDNA™ construct was evaluated in producer cells from panel B. DNA and transcript copy numbers were measured by qPCR and RT-qPCR, respectively, using the indicated probes. Transcript numbers were then normalised to DNA copy numbers for plasmid and dbDNA™ to derive the transcriptional efficiency per DNA molecule. dbDNA™ transcriptional efficiency was then normalised to plasmid, to give relative transcriptional efficiency. Experiments were performed once; error bars represent standard deviation between replicates.

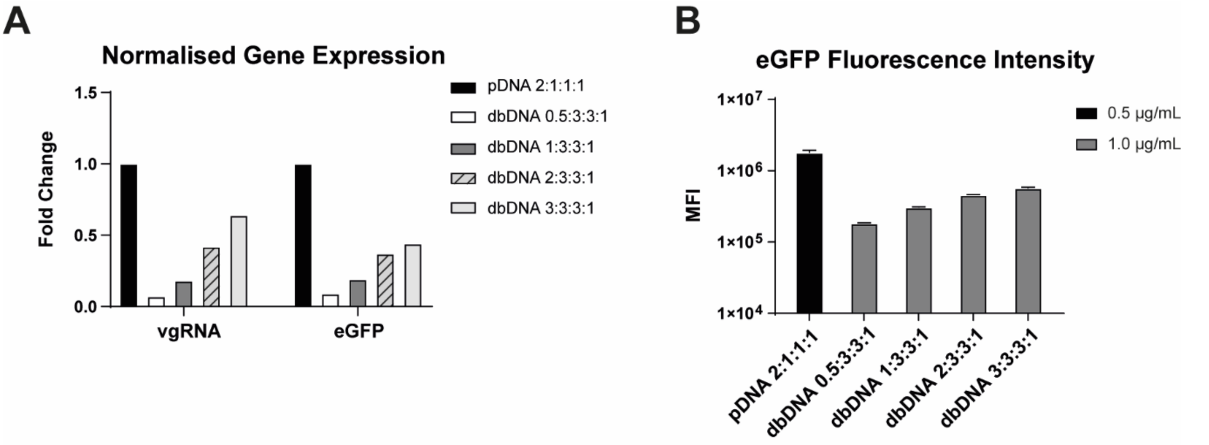

Figure S2. Increasing vgRNA abundance does not translate to enhanced infectivity of dbDNA™ LVV

Viral production cells (VPC) were transfected to produce LVV with 1 µg/mL plasmid (mass construct ratio of 2:1:1:1) or 0.5 µg/mL dbDNA at the indicated molar construct ratios, and cells were harvested 72h post transfection of analysis **(a)** Gene expression measured by RT-qPCR. Probe vgRNA was used to quantify full length viral genomic RNA, and probe eGFP was used for total RNA transcribed from the transfer vector LV-eGFP **(b)** Mean fluorescence intensity (MFI) measured by flow cytometry **(c)** Infectious titre of LVV produced using 1 µg/mL plasmid (mass ratio 2:1:1:1) or 0.5 µg/mL dbDNA at the indicated molar construct ratios. Experiments were performed once; error bars represent standard deviation between replicates.

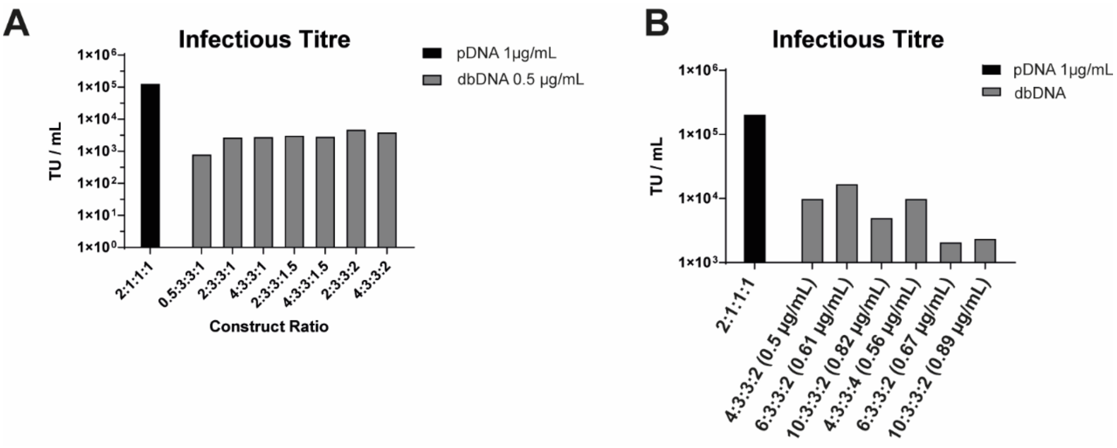

Figure S3. Increasing ratio of transfer vector and VSVg leads to modest improvement in infectivity

(a) VPC were transfected with 0.5 µg/mL dbDNA at the indicated molar construct ratios. Transfer vector was increased from 0.5 to 4, and VSVg was increased from 1 – 2. Standard plasmid conditions were used as a control. Supernatants were harvested at 72h post transfection for analysis of infectious titre by flow cytometry of transduced HEK293T cells (**b)** The ratio of transfer vector and VSVg were further increased to 10 and 4, respectively, as a final test to determine whether significantly increasing the abundance of vgRNA could improve infectivity. To maintain sufficient amounts of accessory proteins under these conditions, the total amount of input dbDNA was slightly increased at higher ratios of transfer vector, as indicated. Supernatants were harvested 72h post transfection for analysis of infectious titre. Experiments were performed once.

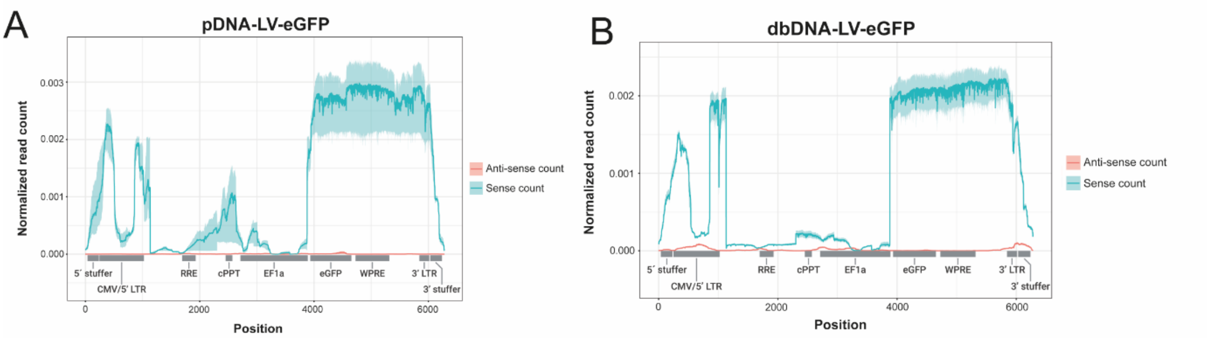

Figure S4. Plasmid and dbDNA transfer vectors display different expression profiles

RNA-sequencing was performed as described in the methods to evaluate the transcriptional profile of (**a**) pDNA and (**b**) dbDNA LV-eGFP transfer vectors. HEK293F cells were transfected in triplicate with 1 µg/mL pDNA or dbDNA and harvested for RNA extraction 48 h post transfection. Data normalization was done by dividing the sense and antisense counts at each nucleotide position by the total number of reads that aligned to the construct reference sequence. Grey bars represent the indicated regions of interest within the sequence. Anti-sense reads are shown in red and sense reads are shown in blue. Areas of blue shading represent variability between samples prepared in triplicate.

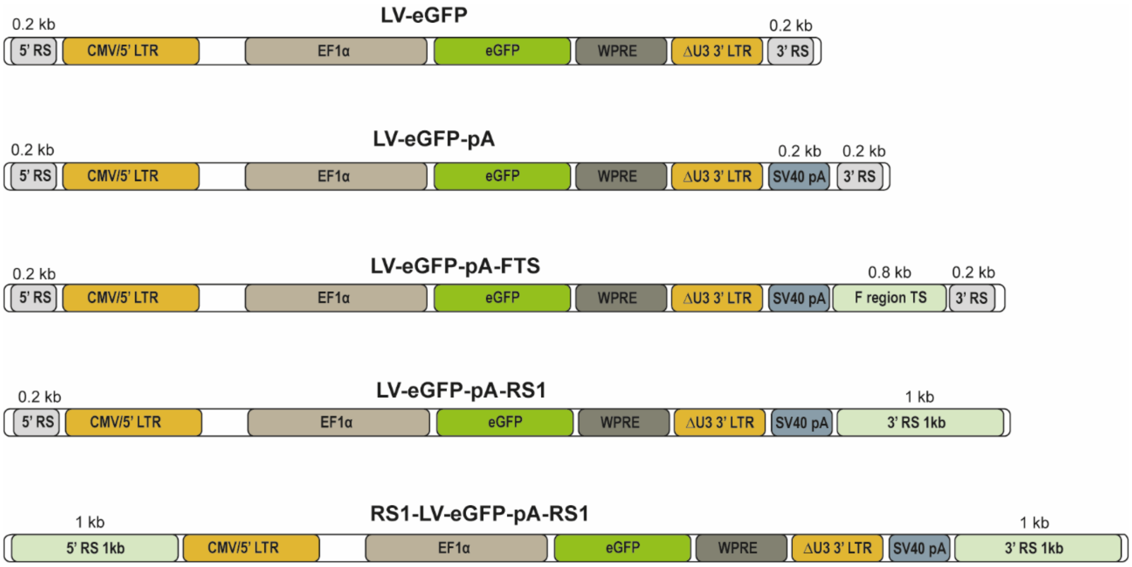

Figure S5. Transfer vector architectures

Shown is a schematic of the dbDNA transfer vector architectures used in this study. RS = random spacer, CMV = Cytomegalovirus promoter, LTR = long terminal repeat, WPRE = woodchuck hepatitis virus posttranscriptional regulatory element, SV40 pA = Simian virus 40 late poly(A)

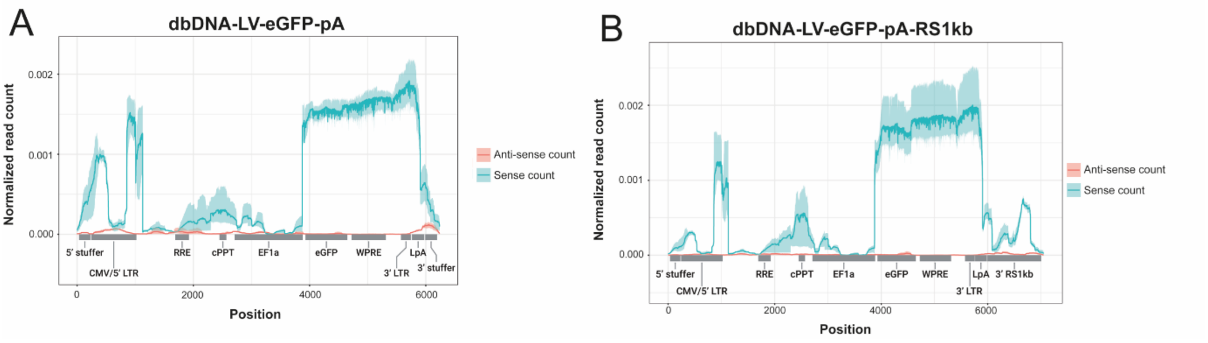

Figure S6. Addition of termination element and downstream spacer improves 3' end processing of vgRNA

RNA-sequencing was performed as described in **Figure S4** to evaluate the transcriptional profile of **(a)** dbDNA-LV-eGFP-pA and (**b)** dbDNA LV-eGFP-pA-RS1 dbDNA transfer vectors. Grey bars represent the indicated regions of interest within the sequence. Anti-sense reads are shown in red and sense reads are shown in blue. Areas of blue shading represent variability between samples prepared in triplicate.

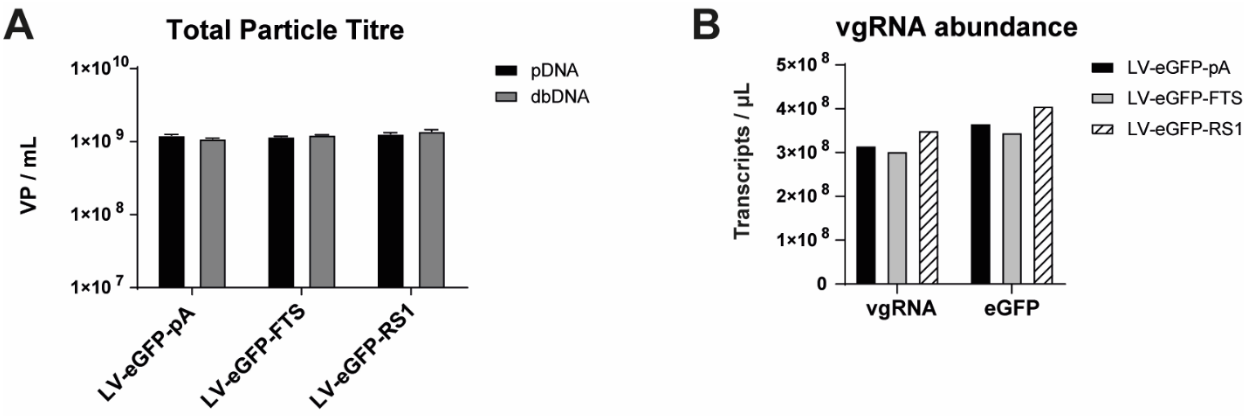

Figure S7. Particle titres and vgRNA abundance are unchanged with addition of poly(A) and RS1kb

LVV productions were carried out using 0.5 µg/mL total dbDNA (molar ratio 4:3:3:4) and the indicated transfer vectors. Supernatants and cells were harvested 72h post transfection for analysis of total particle titre (VP/mL) and vgRNA abundance (**a)** Total particle titre measured by p24 ELISA (**b)** Transfer vector RNA abundance measured by RT-qPCR using probes vgRNA for full length viral genomic RNA and eGFP for total RNA. Experiments were performed once; error bars represent standard deviation between replicates.

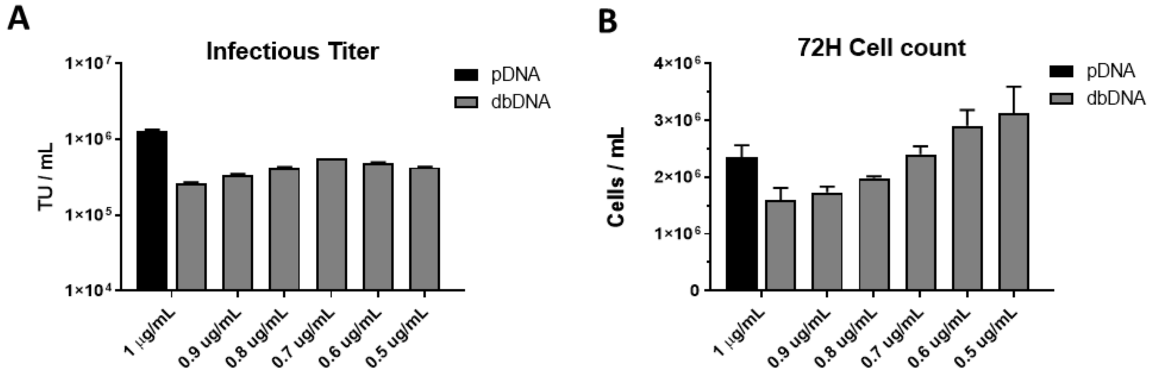

Figure S8. Increasing input dbDNA to 0.7 ug/mL in the context of dbDNA-LV-eGFP-pA-RS1 further boosts infectious titre

VPC were transfected to produce LVV using 0.5 – 1.0 µg/mL total input dbDNA™ and the dbDNA-LV-eGFP-RS1 transfer vector (**a)** Infectious titre (TU/mL) measured by flow cytometry of transduced HEK293 T cells 72 h post transduction (**b)** Cell counts 72 h post transduction. Experiments were performed once; error bars represent standard deviation between replicates.

Table S1. TaqMan primer/probe sets from IDT Technologies

Probe/Primer Name	5' Dye	Sequence	3' Quencher
GAG1 FAM-BHQ1	/56-FAM/	CTGGCCTGTTAGAAACATCAGAAGGCT	/3BHQ_1/
GAG1 FW	None	GAGCTAGAACGATTCGCAGTTA	None
GAG1 RV	None	CTGTCTGAAGGGATGGTTGTAG	None
POL1 FAM-BHQ1	/56-FAM/	TGGTCAGTGCTGGAATCAGGAAAGT	/3BHQ_1/
POL1 FW	None	GTACCAGCACACAAAGGAATTG	None
POL1 RV	None	ATGTTCTTCTTGGGCCTTATCT	None
Rev1 FAM-BHQ1	/56-FAM/	TATCAAAGCAACCCACCTCCCAATCC	/3BHQ_1/
Rev1 FW	None	CAAGGCAGTCAGACTCATCAA	None
Rev1 RV	None	TCTCTCTCCACCTTCTTCTTCT	None
VSVg1 FAM-BHQ1	/56-FAM/	ATTTCCGCTGGTATGGACCGAAGT	/3BHQ_1/
VSVg1 FW	None	CCCAAGAGTCACAAGGCTATTC	None
VSVg1 RV	None	GAGTGAAGGATCGGATGGAATG	None
LTR-P FAM-BHQ1	/56-FAM/	CAGTGGCGCCCGAACAGGGA	/3BHQ_1/
LTR MH531 FW	None	TGTGTGCCCGTCTGTTGTGT	None

LTR MH532 RV	None	GAGTCCTGCGTCGAGAGAGC	None
